# Back to Basics – The Influence of DNA Extraction and Primer Choice on Phylogenetic Analysis of Activated Sludge Communities

**DOI:** 10.1371/journal.pone.0132783

**Published:** 2015-07-16

**Authors:** Mads Albertsen, Søren M. Karst, Anja S. Ziegler, Rasmus H. Kirkegaard, Per H. Nielsen

**Affiliations:** Center for Microbial Communities, Department of Chemistry and Bioscience, Aalborg University, Aalborg, Denmark; Cairo University, EGYPT

## Abstract

DNA extraction and primer choice have a large effect on the observed community structure in all microbial amplicon sequencing analyses. Although the biases are well known, no comprehensive analysis has been conducted in activated sludge communities. In this study we systematically explored the impact of a number of parameters on the observed microbial community: bead beating intensity, primer choice, extracellular DNA removal, and various PCR settings. In total, 176 samples were subjected to 16S rRNA amplicon sequencing, and selected samples were investigated through metagenomics and metatranscriptomics. Quantitative fluorescence *in situ* hybridization was used as a DNA extraction-independent method for qualitative comparison. In general, an effect on the observed community was found on all parameters tested, although bead beating and primer choice had the largest effect. The effect of bead beating intensity correlated with cell-wall strength as seen by a large increase in DNA from Gram-positive bacteria (up to 400%). However, significant differences were present at lower phylogenetic levels within the same phylum, suggesting that additional factors are at play. The best primer set based on *in silico* analysis was found to underestimate a number of important bacterial groups. For 16S rRNA gene analysis in activated sludge we recommend using the FastDNA SPIN Kit for Soil with four times the normal bead beating and V1-3 primers.

## Introduction

Culture-independent methods have revolutionized our understanding of the microbiology of activated sludge communities [1,2] and especially the DNA-based methods for phylogenetic analysis are increasingly applied, due to the exponential decrease in sequencing costs and increased read length [3]. However, you only see what you sequence and only sequence what you can extract and amplify. Hence, the choice of DNA extraction and amplification protocols is pivotal to the outcome of any amplicon sequencing study.

Several studies have investigated the influence of different DNA extraction protocols in activated sludge. However, the comparisons have mostly been made on yield and the ability to obtain reproducible PCR products [4–10]. Only in a few cases have the results been compared to DNA extraction independent methods such as qFISH [11]. Recently, Gou & Zhang [12] used next generation sequencing to evaluate seven DNA extraction kits and showed that methods without bead beating underestimate the presence of bacteria that are typically hard to lyse, such as Gram-positive Actinobacteria. The general conclusions have been that a bead beating step is needed, and the FastDNA spin kit for soil has been recommended due to general high yield and purity [10,12]. Only two papers have investigated the influence of primer choice in activated sludge [13,14] and the often theoretically best primer-sets are applied [15], or they are selected based on recommendations for use in other environments. While the primer choice is known to introduce bias, the input template and specific parameters used in the PCR amplification protocol also influence the observed microbial community [16–18].

Another potential bias on the observed microbial community is the influence of DNA from dead cells [19]. Pre-treatment with propidium monoazide may remove most of the DNA from dead cells by penetrating cells with degraded cell walls and binding to DNA upon photoactivation, which then interferes with the subsequent PCR amplification [20]. However, the effect in activated sludge is presently unknown. In addition to DNA from dead cells, extracellular DNA (eDNA) is a component in the biofilms of activated sludge [21,22]. Currently it is unknown if eDNA is composed of specific sequences, although eDNA has been suggested to be similar to whole genome DNA in *Pseudomonas aeruginosa* biofilms [23]. Interestingly, eDNA has been shown to be in high abundance in the vicinity of selected bacterial groups in activated sludge [22], which might result in a biased view of the microbial community structure.

In this study, we systematically investigated parameters influencing the observed community composition in activated sludge, from sampling and DNA extraction to primer choice and PCR settings, using Illumina MiSeq sequencing of 16S rRNA gene amplicons. Reproducibility was investigated by sampling four separate locations within the same wastewater treatment plant and resolution by sequencing a time-series spanning five months of operation. The effect of short-term storage was investigated by sequencing libraries prepared from samples stored at 4°C for 4 hours, 4°C for 24 hours, or 20°C for 24 hours. In addition, fresh samples were also investigated for the effect of eDNA removal. Instead of surveying a number of different DNA extraction methods, we chose to systematically explore the effect of bead beating intensity and amount of input material, using the commonly applied FastDNA spin kit for soil and MoBio PowerLyzer PowerSoil DNA isolation kit. The effect of primer choice was investigated using three popular primer-sets spanning different variable regions of the 16S rRNA gene; V1-3 (used by Human microbiome project [24]), V3-4 (recommended based on theoretical analysis [15], and the V4 (used by the Earth microbiome project [3]). To explore the effect of the PCR settings, we varied the amount of template, number of cycles and annealing temperature. For comparison, selected samples were also investigated by PCR-free metagenomics and stranded metatranscriptomics. Finally, the results were qualitatively compared with qFISH, using 23 oligonucleotide probes covering many of the important and abundant bacterial groups in activated sludge [25].

## Materials and Methods

### Sampling

Activated sludge was sampled from aeration tanks at Aalborg West WWTP (57.049422° N, 9.864735° E) and Aalborg East WWTP (57.036973° N, 10.016569° E) in Denmark from October 2012 through March 2013. Sampling was carried out with the permission of Aalborg Forsyning, Kloak A/S. The plants primarily receive domestic wastewater (avg. 195 000 PE and 45 000 PE), and employ nitrogen and enhanced biological phosphorus removal. The aeration tanks were well mixed, and 1 L activated sludge was sampled 1 m below the surface. Sample material was transported on ice and processed within 4 h. The samples were homogenized (1 min, 1650 rpm) using a mortar-pestle, glass/teflon tissue homogenizer (30 mL) mounted on a Heidolph RZR 2020 (Heidolph, Germany) and stored as 2 mL aliquots at -80°C. To investigate the heterogeneity of the aeration tanks, four samples were obtained from different locations in the aeration tanks. To simulate the effect of longer transport time before processing, a subsample was stored at 20°C and shaken at 100 RPM for 24 h and another stored at 4°C for 24 h before processing. Subsamples of all samples were fixed for qFISH analysis [26]. Total solid (TS) content was measured in duplicate for all samples [27].

### Removal of DNA from dead cells and extracellular DNA

Fresh activated sludge samples were spiked with extracted *E*. *coli* DNA in 0 to 50% concentrations relative to the TS content of the sludge. Each sample was then divided into six 2 mL tubes, and three were treated with propidium monoazide (PMA). Samples not treated with PMA were kept on ice until DNA extraction. PMA (Biotium, USA) was added to a final concentration of 100 μM, and the samples were incubated in the dark for 10 min with occasional shaking. After incubation, the samples were subjected to a strong visible light (650 W halogen light bulb) for 10 min at a distance of approx. 20 cm, while keeping the samples on ice.

### DNA extraction

The standard protocols for DNA extraction with FastDNA Spin kit for soil (MP Biomedicals, USA) and PowerLyzer PowerSoil DNA isolation kit (MoBio, USA) were used with the following exceptions. Samples were thawed at room temperature, and varying amounts of biomass (0.9–2.2 mg TS) were spun down (5 min at >10,000 x g) and re-suspended in the respective lysis or bead solutions before extraction. Bead beating was performed on a FastPrep FP120 (MP Biomedicals, USA) at different intensity settings (20–400 s at 4–6 m/s), following general operation recommendations. All extraction conditions were tested in triplicate.

Purity of the extracted DNA was evaluated spectrophotometrically with Nanodrop1000 using A_260/230nm_ and A_260/280nm_ (Thermo Fisher Scientific, USA). The quality of the extracted DNA was evaluated with agarose gel electrophoresis, using the Tapestation 2200 and Genomic DNA screentapes (Agilent, USA). Finally, the concentration was measured fluorometrically with Quant-iT HS DNA Assay (Thermo Fisher Scientific, USA) on an Infinite M1000 PRO (Tecan, Switzerland).

### 16S rRNA amplicon sequencing

The procedure for bacterial 16S rRNA amplicon sequencing targeting the V1-3, V3-4, and V4 variable regions was modified from Caporaso *et al*. [3]. Amplicon library PCR was performed on all replicate extractions independently. Ten ng of extracted DNA was used as template and the PCR reaction (25 μL) contained dNTPs (400nM of each), MgSO_4_ (1.5 mM), Platinum Taq DNA polymerase HF (2mU), 1X Platinum High Fidelity buffer (Thermo Fisher Scientific, USA), and a pair of barcoded library adaptors (400 nM). V1-3 primers: 27F AGAGTTTGATCCTGGCTCAG and 534R ATTACCGCGGCTGCTGG. V3-4 primers: 314F CCTACGGGNGGCWGCAG and 805R GACTACHVGGGTATCTAATCC. V4 primers: 515F GTGCCAGCMGCCGCGGTAA and 806R GGACTACHVGGGTWTCTAAT. Thermocycler settings for V1-3 and V4 PCR: Initial denaturation at 95°C for 2 min, 30 cycles of 95°C for 20 s, 56°C for 30 s, 72°C for 60 s and final elongation at 72°C for 5 min. Thermocycler settings for V3-4 PCR: Initial denaturation at 95°C for 2 min, 30 cycles of 95°C for 20 s, 50°C for 30 s, 72°C for 30 s and final elongation at 72°C for 5 min. The effect of different annealing temperatures (52-56-58°C), template amounts (1-5-50 ng DNA) and the number of PCR cycles (25-30-35 cycles) was tested, all performed with V1-3 primers. All PCR reactions were run in duplicate and pooled afterwards. The amplicon libraries were purified using the Agencourt AMpure XP bead protocol (Beckmann Coulter, USA) with the following exceptions: the sample/bead solution ratio was 5/4, and the purified DNA was eluted in 33 μL nuclease-free water. Library concentration was measured with Quant-iT HS DNA Assay (Thermo Fisher Scientific, USA) and quality validated with a Tapestation 2200, using D1K ScreenTapes (Agilent, USA). Based on library concentrations and calculated amplicon sizes, the samples were pooled in equimolar concentrations and diluted to 4 nM. The library pool was sequenced on a MiSeq (Illumina, USA), using a MiSeq Reagent kit v3 (2x300 PE) following the procedure in *Caporaso et al*. [3], with the exception of 10% PhiX control library (Illumina, USA) spike-in and final library loading concentration of 20 pM.

### Amplicon bioinformatic processing

All sequenced sample libraries were subsampled to 50,000 raw reads and screened for PhiX contamination using bowtie2 v. 2.1.0 [28] with standard settings and all matching reads removed. The potential PhiX contamination is due to the use of an un-indexed PhiX as a quality control, which can result in index carryover from nearby clusters with indexes.

Forward and reverse reads from the V4 amplicons were trimmed for quality by truncating reads when the median quality of the reads dropped below a Phred score of 20. The trimmed V4 reads were merged using FLASH v. 1.2.7 [29]. For the V1-3 and V3-4 amplicons, only the first 225 bp of each amplicon were used as the quality of the reverse reads from some sequencing runs prevented a decent merging of reads.

The reads were dereplicated and formatted for use in the UPARSE workflow [30]. The dereplicated reads were clustered, using the usearch v. 7.0.1090-cluster_otus with default settings. OTU abundance was estimated using the usearch v. 7.0.1090-usearch_global with-id 0.97. Taxonomy was assigned using the RDP classifier [31] as implemented in the parallel_assign_taxonomy_rdp.py script in QIIME [32], using MiDAS taxonomy version 1.20 [33], which is based on the SILVA taxonomy [34].

The results were analysed in R [35] through the Rstudio IDE (http://www.rstudio.com/). To facilitate effortless analysis and visualization of the amplicon data, we build the R package ampvis v.1.9.2 (https://github.com/MadsAlbertsen/ampvis) which builds on the R packages phyloseq [36], ggplot2 [37], reshape2 [38], dplyr [39], vegan [40], knitr [41], Biostrings [42], data.table [43], DESeq2 [44], ggdendro [45] and stringr [46].

### Bioinformatic analysis

Principal component analysis (PCA) was conducted, using vegan [40] with square root transformed OTU counts. Significance of treatments was tested, using the envfit parametric test on the first two principal components and on the Bray-Curtis dissimilarity matrix, using adonis as implemented in vegan. In addition, sample clustering was visualized using hierarchical clustering of the Bray-Curtis dissimilarity matrix. OTUs in differential abundance between treatments were identified, using DESeq2 [44] with test = “wald” and fitType = “parametric”.

### Metagenomics

Three replicate DNA extractions used for amplicon sequencing were selected for metagenome sequencing and prepared according to the recommendations in the Illumina TruSeq PCR free protocol (Illumina Inc.). The prepared libraries were paired-end sequenced, using 2x301 bp MiSeq Reagent kits v3 on an Illumina MiSeq. The reads were trimmed, using CLC genomics workbench v.7.03 (CLCbio, Qiagen) by requiring a minimum Phred score of 20, a minimum length of 200 bp and removing any adaptors, if found. In addition, the reverse read was discarded due to relatively low quality, compared to the forward read and in order not to bias the subsequent count-based analysis. The trimmed metagenome reads were mapped to the MiDAS database version 1.20 [33], using the map reads to reference function in CLC genomics workbench v. 7.03, requiring 95 percent similarity over the full read length and random assignments of reads, which mapped to two sequences equally well. The results were exported as.csv files, imported to R and converted to phyloseq objects for easy manipulation and visualization. See the online documentation for the exact workflow applied.

### Metatranscriptomics

Three replicate samples were subjected to RNA extraction, using the RiboPure-Bacteria Kit (Thermo Fisher Scientific, USA) according to the protocol, except that the input was 2.2 mg TS activated sludge biomass, and bead beating (160s at 6 m/s) was performed with FastPrep FP120 (MP Biomedicals, USA). Extracted total RNA purity was evaluated with Nanodrop1000 (Thermo Scientific Fisher, USA), quality with Tapestation 2200, using High Sensitivity screentapes (Agilent, USA) and concentration, using Qubit RNA BR Assay Kit (Thermo Scientific Fisher, USA). The extracted RNA was used for library preparation, using the TruSeq stranded mRNAseq protocol (Illumina Inc., USA) according to the manufacturer’s recommendations and sequenced, using 2x75bp MiSeq Reagent kits v3 on an Illumina MiSeq. The reads were trimmed, using CLC genomics workbench v.7.03 (CLCbio, Qiagen), requiring a minimum Phred score of 20 and a minimum length of 75 bp. In addition, the reverse reads were discarded in order not to bias the subsequent count based analysis. The trimmed metatranscriptome reads were mapped to the MiDAS database version 1.20 [33], using the map reads to reference function in CLC genomics workbench v.7.03, requiring 95 percent similarity over the full read length and random assignments of reads, which mapped to two sequences equally well. The results were exported as.csv files, imported to R and converted to phyloseq objects for easy manipulation and visualization. See online documentation for the exact workflow applied.

### Theoretical primer coverage

The theoretical coverage of each primer-set was calculated using the TestPrime 1.0 function implemented at www.arb-silva.de [15] against the SILVA SSU r119 database. The analysis was conducted with 0, 1, 2, or 3 mismatches for each primer-set and otherwise default settings.

### Quantitative FISH

Quantitative FISH (qFISH) was conducted as described in Morgan-Sagastume *et al*. [26] and Nielsen *et al*. [25] with an extensive set of 23 oligonucleotide probes that covers most of the diversity usually found in Danish wastewater treatment plants with nutrient removal.

## Results

All experiments were conducted as independently sampled biological triplicates and had at least 25,000 non-unique quality filtered sequences per sample after sequencing and bioinformatic processing. If nothing else is stated, the optimized DNA extraction and amplification protocol was applied (four times the kit-recommended bead beating and V1-3 primers).

### Reproducibility, resolution, and short-term storage

**Fig 1:**
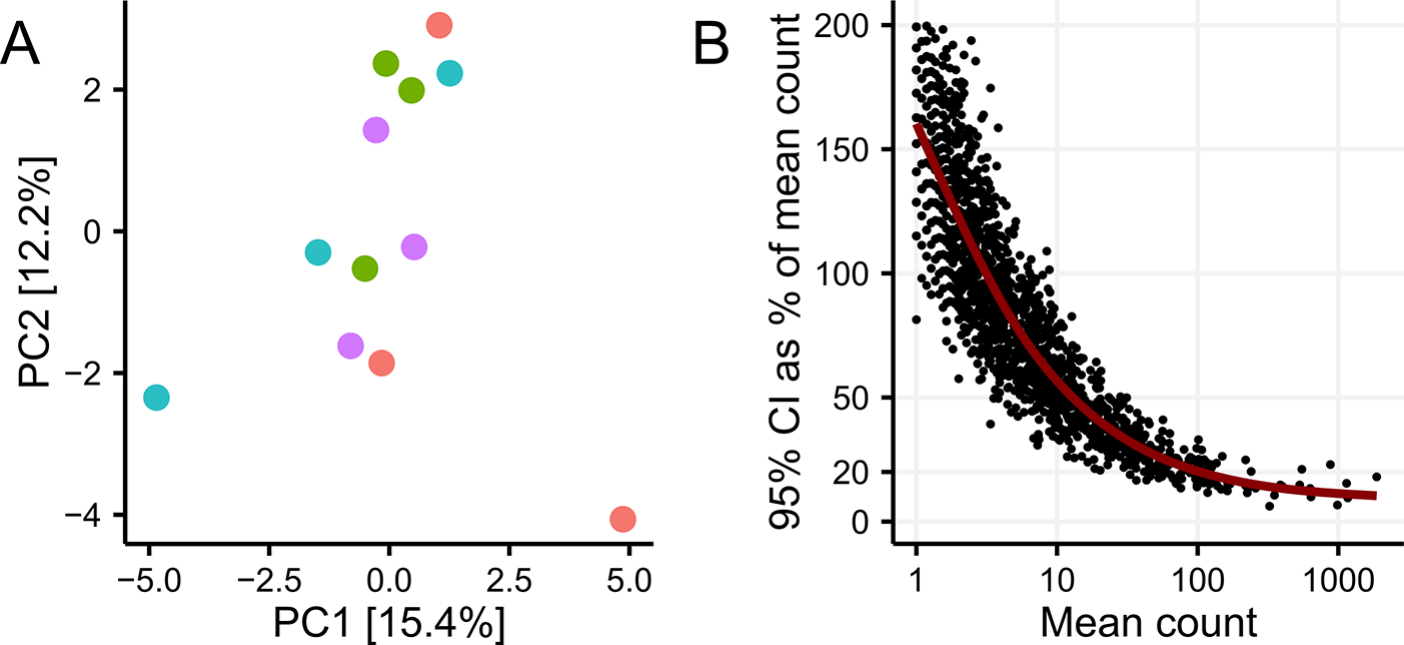
Effect of sampling. (A) No clustering by sampling site was seen in the PCA analysis of square root transformed OTU abundances (p_adonis_ = 0.53, n_seq_ = 17000, n_sample_ = 12). (B) OTU level variation when sequencing three biological replicates as a function of sequencing depth. Variation was measured as the 95% confidence interval in percentage of the mean OTU read count (n_seq_ = 32000, n_sd_ = 11, n_se_ = 3).

To investigate the reproducibility of the sampling at the wastewater treatment plant, two different aeration tanks were sampled in both ends of the basins. There was no significant effect of sampling position (p_adonis_ = 0.1, n = 12, Fig 1A), indicating that the aeration basins were completely mixed and sampling reproducible if handled properly. The variation within the biological replicates was small enough to distinguish between samples taken only weeks apart (p_adonis_ = 0.006, n = 9, S1 Fig). At OTU level, the variation was highly dependent on sequencing depth. Using three biological replicates, the variation in estimation of the mean decreased as a function of sequencing depth, until it reached a 95% confidence interval of approximately 20% of the mean, above 100 reads (Fig 1B, n_sd_ = 11, n_se_ = 3).

In order to mimic the process of sampling and short-term storage from the wastewater treatment plant to the laboratory, samples were subjected to incubation at either 4 h at 4°C, 24 h at 4°C or 24 h at 20°C before being deposited in -80°C storage and subsequently sequenced. While there was a small effect of the short-term storage methods (p_adonis_ = 0.01, n = 9) on the overall community composition, it was small, compared to the variation between time-points months apart, but could potentially influence the conclusions drawn from samples within a weekly timeframe (S2 Fig).

### Extracellular DNA

**Fig 2:**
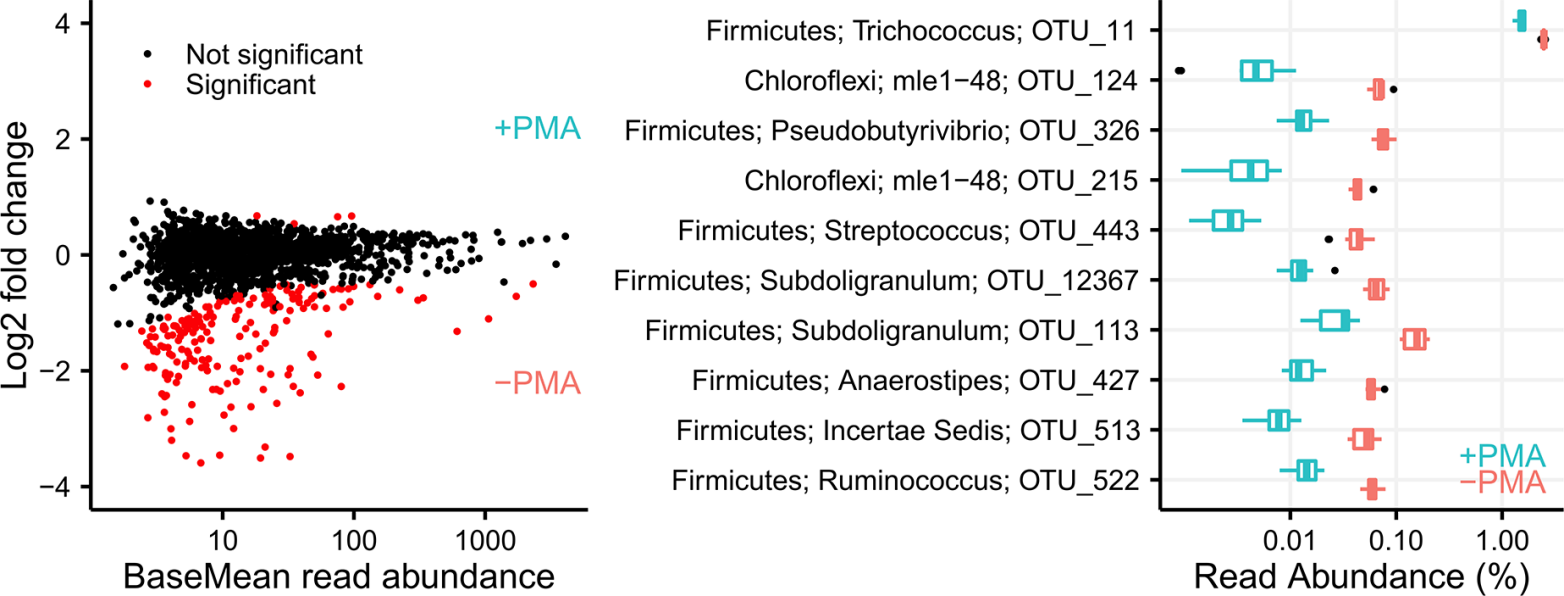
Effect of PMA treatment on individual OTUs. (A) OTUs determined to be in significant different abundance after PMA treatment using DESeq2. Only species with a p_adj_ < 0.001 are visualized as being in significant different abundance. (B) The 10 OTUs with the lowest p-value. For all OTUs a phylum and genus classification is shown along with the OTU number.

The effect of removing extracellular DNA was investigated by treating samples with PMA. The PMA treatment removed all spiked-in *E*. *coli* DNA (S3 Fig), showing that PMA was able to remove naked DNA. The PMA treatment significantly changed the overall community profile (p_adonis_ = 0.001, n = 24) and reduced alpha diversity, measured as the number of observed OTUs (p_t.test_ = 7.1e^-5^, n = 24). In total, 197 of 1643 OTUs were significantly affected by the PMA treatment (p_adj(DESeq2)_ < 0.001, n = 24; Fig 2). The most significantly changed OTU was OTU_11 related to the genus *Trichococcus* within the phylum Firmicutes (p_adj(DESeq2)_ = 5.8e^-81^, n = 24). Compared to the time series, the effect of the PMA treatment was large, although treated and untreated samples from the same time point were more similar to each other than the rest of the time series (S3 Fig).

### Amount of input material

The effect of different amounts of input material was investigated by using 0.9, 2.2, or 22 mg of activated sludge (TS) as input to the DNA extraction protocol. The 0.9 and 2.2 mg samples clustered together, while the samples extracted using 22 mg of activated sludge as input clustered separately (S4 Fig).

### Bead beating

**Fig 3:**
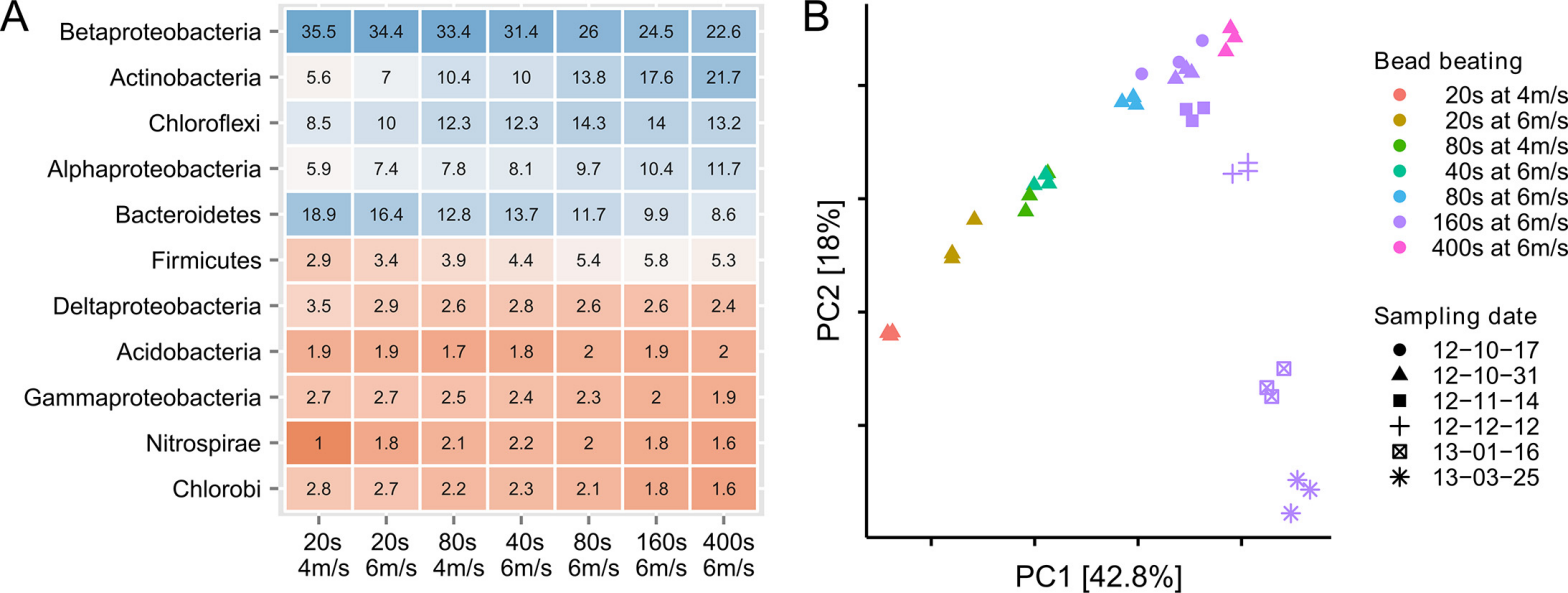
The effect of bead beating on the observed microbial community composition. (A) Percentage read abundance of the 11 most abundant phyla as function of bead beating intensity (Proteobacteria are show at class level). The data is visualized as a table with the underlying colors visualizing the changes. (B) Principal component analysis of the samples extracted with different bead beating intensities compared to the samples taken at different dates, but extracted with the same bead beating settings (160 s at 6 m/s).

To explore the effect of bead beating on the observed community composition, the bead beating intensity was varied around the recommended setting (40 s at 6 m/s) in the FastDNA SPIN Kit for Soil protocol. Increased bead beating had a dramatic influence on the observed community composition (Fig 3A). Compared to the time series samples, the effect of bead beating was larger than the effect of sampling 5 months apart (Fig 3B). However, despite the large effect of bead beating, it was still possible to distinguish samples from different wastewater treatment plants (S5 Fig).

**Fig 4:**
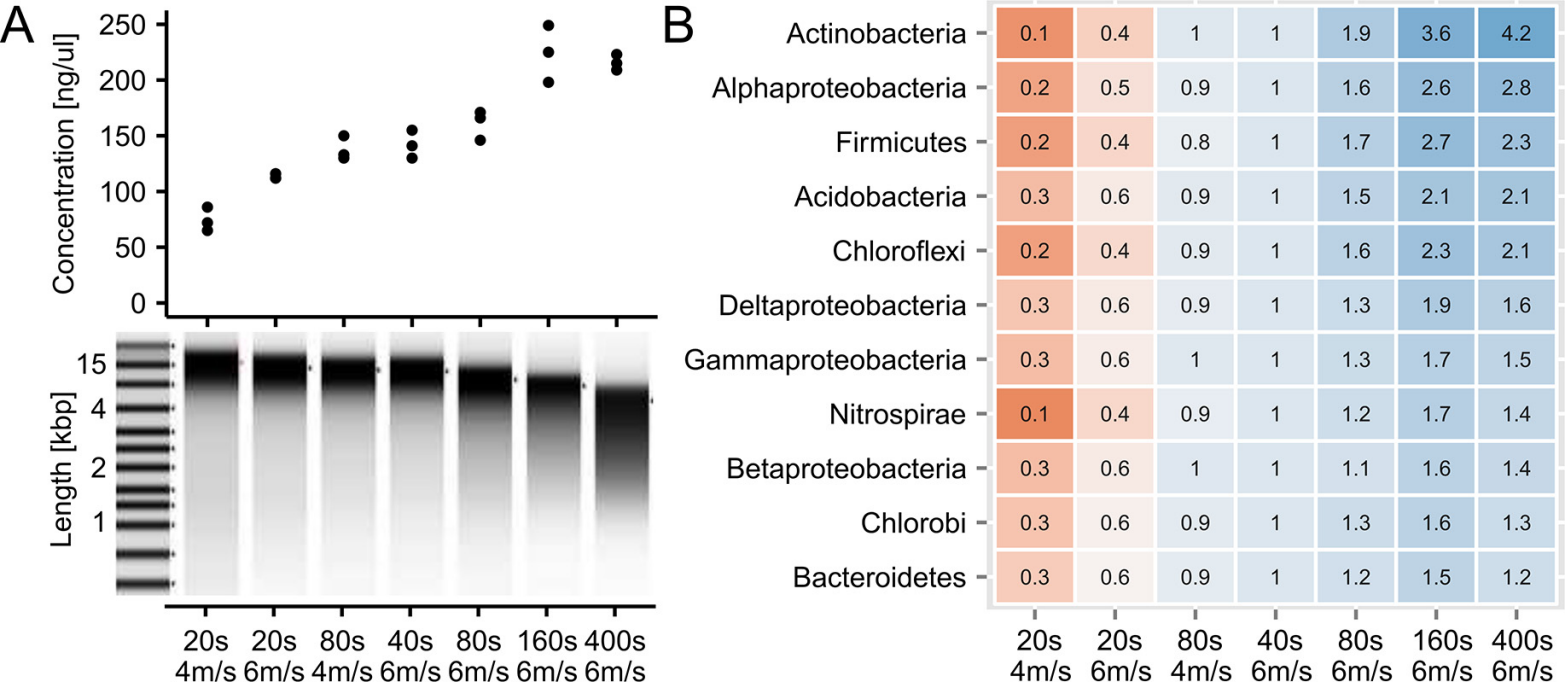
The effect of bead beating on DNA yield, integrity and phylogenetic composition at phylum level. (A) The increase in yield as a function of bead beating intensity. While yield increased with bead beating, the DNA also became more fragmented. (B) Comparison of absolute phylum level differences as function of bead beating intensity. The relative read abundances were scaled by the DNA yield to obtain absolute counts and normalised relative to the standard settings (40 s at 6 m/s) to facilitate direct comparison between different phyla. The data is visualized as a table with the underlying colors visualizing the changes.

The change in community composition with increased bead beating could be an effect of degradation of DNA from fragile bacterial groups (Fig 3A). However, by accounting for yield, all phylum level bacterial groups increased in absolute numbers (Fig 4). The effect of increased bead beating was highest for bacteria associated with the Gram-positive phylum Actinobacteria with an average of four times higher absolute abundance, compared to the standard settings, and a relative increase from 10.0% to 21.7% of all reads (Fig 3A). The large effect of bead beating was also observed using the V4 primers (S6 Fig) and another DNA extraction kit (MoBio, S7 Fig). The phylum level differences in extraction efficiencies were also seen at order level, although notable differences were present within each group (S8 Fig). For example, within the abundant orders of Actinobacteria, the absolute change varies from 2.7 to 6.3 fold and within Alphaproteobacteria from 1.2 to 3.7 fold (S9 Fig).

### Primer choice

**Fig 5:**
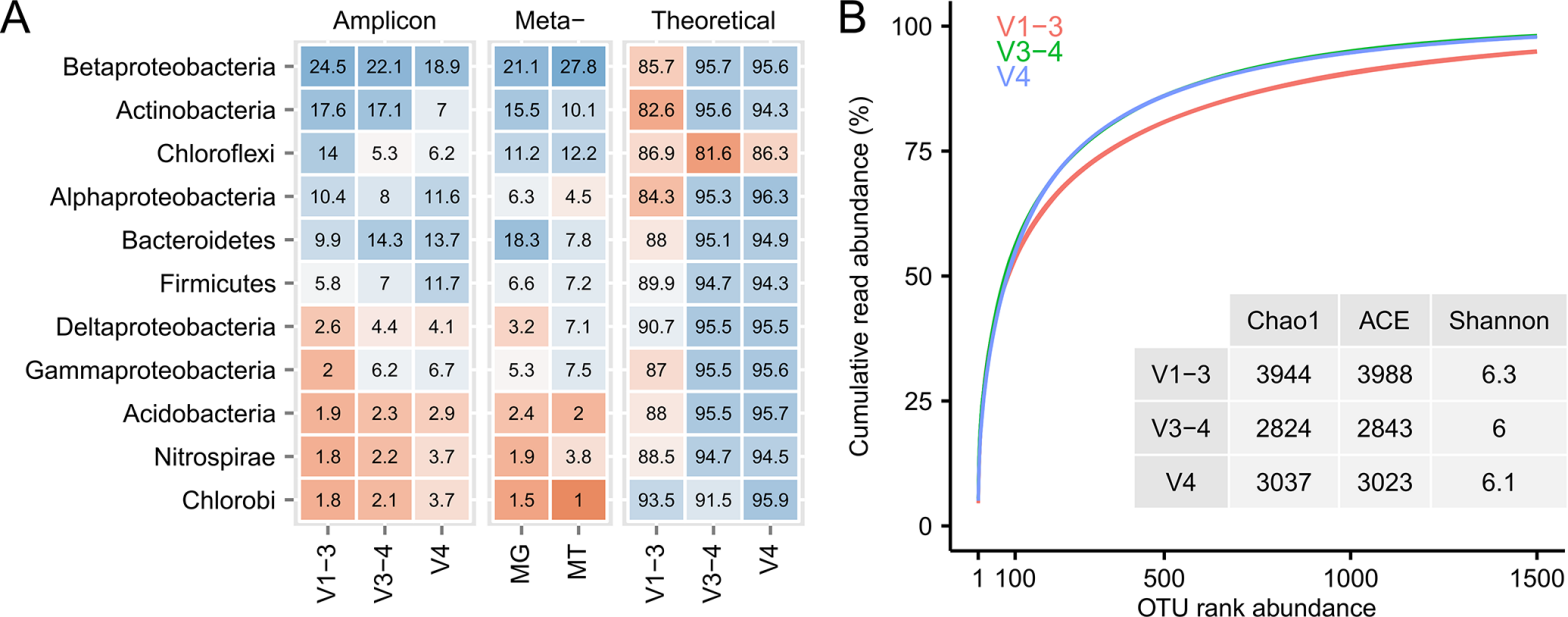
The effect of primer choice on the observed phylogenetic composition. (A) Comparison of the phylogenetic composition at phylum level using three different sets of primers (V1-3, V3-4, V4), PCR-free metagenomics (MG), stranded metatranscriptomics (MT), and the theoretical primer-set coverage with 1 mismatch allowed. The data is visualized as a table with the underlying colors visualizing the changes. (B) Comparison of alpha-diversity between the different sets of primers using diversity indices and rank abundance curves.

The three sets of primers showed very different community profiles, but similar diversity indices (Fig 5). For direct comparison, we sequenced PCR-free metagenomes (Fig 5, MG) from the same DNA as was used for the amplicon samples. In general, the dominant phyla in the metagenomes were also dominant in the amplicon samples. However, the V1-3 primers seemed better at capturing bacteria from the Actinobacteria and Chloroflexi, while Gammaproteobacteria were underestimated. The V4 primers seemed furthest from the observed metagenome diversity. While much of the difference observed between the primers is due to different amplification efficiencies, some orders were not amplified at all (S10 Fig). For example, the V1-3 primers successfully amplified Xanthomondales within the Gammaproteobacteria, but failed to amplify both Pseudomondales and Alteromondales from the same class.

For qualitative comparisons we also sequenced stranded transcriptomes, which showed that the bacterial groups abundant in the DNA-based methods were also the most abundant in the transcriptomes. In addition, the qFISH analysis of a number of important probe-defined bacterial groups (S1 File) also showed a high abundance of Actinobacteria and Chloroflexi.

The theoretical analysis of the primer-set coverage showed that the V4 primers had the best coverage of both Bacteria and Archaea, while the V1-3 primers had the lowest coverage (Fig 5).

### PCR settings

**Fig 6:**
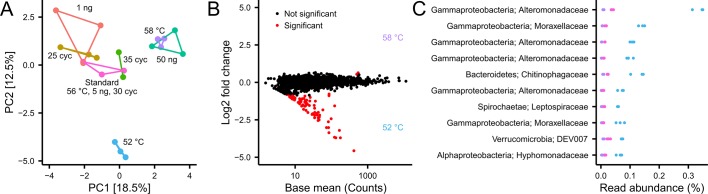
The effect of PCR settings on observed microbial community. (A) Principal component analysis of all PCR settings tested. (B) Differential abundance analysis of OTUs as an effect of different annealing temperatures. (C) The 10 most significantly differential abundant OTUs as a result of a change in annealing temperature.

The effect of different PCR settings was tested by varying the amount of template DNA, annealing temperature, and the number of cycles. There was a significant effect of different PCR settings on the overall community composition (Fig 6, n = 20, p_adonis_ = 0.001). However, for both the amount of template and the number of cycles only small changes were seen on the individual OTUs. Interestingly, lowering the annealing temperature from 58°C to 52°C increased the abundance of some OTUs that were barely detected at 58°C, while not changing the abundance of the majority of OTUs (Fig 6).

## Discussion

Many studies have evaluated parameters that influence the observed microbial communities in DNA-based studies, but often only few parameters are evaluated, and hence their individual contributions are difficult to assess. Here we focused on the major sources of variation from sampling to DNA extraction and primer choices in order to evaluate their relative contribution to the observed variance and hence their influence on the resolution power of the analysis.

Even though wastewater treatment plants consist of large process tanks that may seem heterogeneous, we did not observe any significant effect when sampling in four separate locations of the aeration basins. The resolution with three replicates enables comparisons of samples on a weekly timescale within the same treatment plant. However, our results indicate that the weekly resolution is lost if the samples are not transported appropriately. Shipping samples at room temperature with overnight delivery should be avoided if a weekly resolution is warranted.

The influence of DNA from dead cells and potential extracellular DNA was investigated through pre-treatment of the samples with PMA that can penetrate disrupted cell walls and bind to DNA through photoactivation. PMA treatment is rarely applied in environmental samples, but has gained a lot of interest in clinical samples, where it is essential to get viable cell counts [19]. The PMA treatment had a large effect on the beta-diversity when seen in the context of time series samples, but still enabled clustering with untreated samples from the same time-point. The most significantly affected OTU belonged to the genus *Trichococcus*, which has been found to be abundant in the wastewater influent [47], and this might indicate that a substantial proportion of *Trichococcus* arriving via the influent cannot survive in the treatment plant. However, even though the PMA treatment seems to alter the abundance of some community members, the majority of OTUs were unaffected. Considering that PMA treatment needs to be conducted on fresh samples, we think the additional effort to process every sample when sampled outweighs the additional benefit of removing a few potentially dead cells. However, PMA treatment might be useful in environments where the carbon turnover is less than in wastewater treatment plants.

DNA extraction in activated sludge is often carried out by the FastDNA Spin Kit for Soil [10,12,48]. Like many other methods, it relies on chemical lysis in combination with physical disruption, using bead beating. The bead beating intensity recommended by the manufacturer (6 m/s for 40 s) was insufficient in extracting DNA from many bacterial groups that are known to be difficult to lyse, e.g. Gram-positive bacteria. The increase in recovery reached a plateau for most bacterial groups at 6 m/s for 160 s. Further increase of bead beating led to a marked reduction in DNA integrity and only increased the recovery of Actinobacteria. It is unknown if a substantial proportion of bacteria remains undetected, however a qualitative comparison with the qFISH results suggests that DNA from the majority of the community members is extracted. In addition, the plateau of DNA yield and the abundance of most bacterial species after bead beating for 160 s at 6 m/s indicate that the limit was reached regarding what can be extracted by the applied method. Compared to Gram-negative bacteria, the Gram-positive species, in general, seemed more difficult to extract DNA from, although there were great differences even within orders of the same phylogenetic class. This points to other sources of variation such as micro-colony strength preventing extraction of DNA from specific microbial groups. The variation in the observed microbial composition from the bead beating experiment was larger than the variation in samples taken 5 months apart, which highlights the need for standardized DNA extraction methods, as echoed by all previous papers investigating the topic. In activated sludge, four times the normal bead beating duration (i.e. 4x40 s at 6 m/s) seems to be an optimal compromise between maintaining DNA integrity and representative extraction of bacteria that are difficult to lyse. The effect of increased bead beating was not only observed with the FastDNA Spin Kit for Soil, but also using the MoBio PowerLyzer PowerSoil DNA isolation kit, which is commonly applied in many different environments. While similar results were obtained regarding the microbial community composition, the yield was significantly lower in the MoBio PowerLyzer PowerSoil DNA isolation kit (data not shown).

The effect of primer choice was investigated using three popular primer-sets spanning different variable regions of the 16S rRNA gene; V1-3 (used by the Human microbiome project [24]), V3-4 (recommended based on theoretical analysis [15]) and V4 (used by the Earth microbiome project [3]). As there is no absolute truth and the primers target different variable regions, the evaluation of the “best” primer-set relies on a qualitative evaluation. The three primer sets resulted in very different community structures, even though they were applied to the exact same extracted DNA. Only the V1-3 primers captured a reasonable number of Chloroflexi, which are often abundant in treatment plants and responsible for much of the dynamic changes and potential settling problems [49]. However, it was particularly bad at detecting specific orders within the Gammaproteobacteria, although detection of the functional important genus *Ca*. Competibacter [50] seemed fine. The V4 primer set has often been highlighted as a good general primer set as it has one of the highest phylogenetic coverages based on *in silico* analysis [15]. However, in activated sludge, it greatly underestimates the abundance of Chloroflexi and Actinobacteria, which are often the predominating members of the community. Interestingly, the V1-3 primer-set performs poorly in the *in silico* test, but seems quite good when applied *in situ*. For activated sludge, we recommend the use of the V1-3 primer-set as it has a good overall agreement with the PCR-free metagenomes and captures the highest percentage of the Chloroflexi.

Finally, we tested the impact of template concentration, number of cycles, and annealing temperature during the PCR step on the observed community composition. Although some studies have shown that the number of cycles has a profound effect on the relative abundance of community members in simple communities [16] we did not observe any effect, which might be attributed to a more complex community, where template reannealing is limited. Similarly, we did not observe any major differences due to different template concentrations, although recent studies have shown that a higher template concentration decreases the variance [18]. Lowering the annealing temperature resulted in amplification of several OTUs that were undetected at higher annealing temperatures; interestingly, there did not seem to be any negative effect of lowering the annealing temperature. While the experimental design employed in this study indicate the effect of individual parameters, some effects might be hidden due to interaction among parameters. These could be explored in future studies using a factorial experimental design of selected variables.

## Conclusion

For 16S rRNA amplicon analysis in activated sludge, we recommend the use of the FastDNA spin kit for soil with four times the standard bead beating (160s at 6 m/s) and the V1-3 primer set. Complete step-by-step protocols can be found at www.midasfieldguide.org. Although we only investigated the influence of the selected parameters in activated sludge, similar effects might be expected in other environmental samples. We also recommend that the amplicon analysis be used with the MiDAS curated taxonomy [33] to provide a solid joint foundation for the study of microbial ecology of the activated sludge process and related treatment processes.

## Supporting Information

S1 Fig Resolution using time-series data. (A) PCA analysis of square root transformed OTU abundances. (B) Hierarchical clustering using Bray-Curtis dissimilarity. The variation within the biological replicates is small enough to distinguish between samples taken only weeks apart (p_adonis_ = 0.006, n = 9).
(TIFF)

S2 Fig Effect of storage method. (A) PCA analysis of square root transformed OTU abundances. (B) Hierarchical clustering using Bray-Curtis dissimilarity. (C and D) The effect of storage seen in the context of the time series samples. While there was a significant effect of the short-term storage methods (p_adonis_ = 0.01, n = 9) on the overall community composition, it was small compared to the variation between samples months apart, but could potentially influence the conclusions drawn from samples within a weekly time frame.
(TIFF)

S3 Fig Effect of PMA treatment on alpha and beta diversity. (A) PMA treatment removed all spiked-in DNA from *E*. *coli*. In the subsequent analysis, *E*.*coli* OTUs were removed and samples subsampled to the same number of sequences (17000) to facilitate comparisons using the whole dataset. (B) Impact of PMA tratment on the observed number of OTUs (p_t.test_ = 7.2e^-5^, n = 24). (C) PCA analysis of square root transformed OTU abundances. (D) Hierarchical clustering using Bray-Curtis dissimilarity. The PMA treatment significantly changed the overall community profile (p_adonis_ = 0.001, n = 24). (E and F) The effect of PMA treatment seen in the context of the time series samples.
(TIFF)

S4 Fig Effect of the amount of input material. (A) PCA analysis of square root transformed OTU abundances. (B) Hierarchical clustering using Bray-Curtis dissimilarity.
(TIFF)

S5 Fig Time series vs. bead beating and another WWTP. While the resolution is lost within months using different bead beating settings, the two different WWTPs can still be separated (AAW and AAE).
(TIFF)

S6 Fig Effect of bead beating using the V4 primers. Absolute abundance was calculated by accounting for DNA yield and then normalised to the standard bead beating setting (40s at 6 m/s) to facilitate comparison between phyla (Proteobacteria are show at class level).
(TIFF)

S7 Fig Effect of bead beating using the MoBio PowerLyzer PowerSoil DNA isolation kit. (TIFF)

S8 Fig Effect of bead beating at order level (relative). Both phylum and order level taxonomic classifications are shown (Proteobacteria are shown using classes instead).
(TIFF)

S9 Fig Effect of bead beating at order level (absolute). Absolute abundance was calculated by accounting for DNA yield and then normalised to the standard bead beating setting (40 s at 6 m/s) to facilitate comparison between groups. Both phylum and order level taxonomic classifications are shown (Proteobacteria are shown using classes instead).
(TIFF)

S10 Fig Effect of primers at order level. Both phylum and order level taxonomic classifications are shown (Proteobacteria are shown using classes instead). MG = Metagenome; MT = Metatranscriptome.
(TIFF)

S1 File qFISH results. (PDF)
