# Supplementary figures and images for: Back to Basics – The Influence of DNA Extraction and Primer Choice on Phylogenetic Analysis of Activated Sludge Communities
Source: PLoS One. 2015 Jul 16;10(7):e0132783. doi: 10.1371/journal.pone.0132783 (PMC4504704; doi:10.1371/journal.pone.0132783)

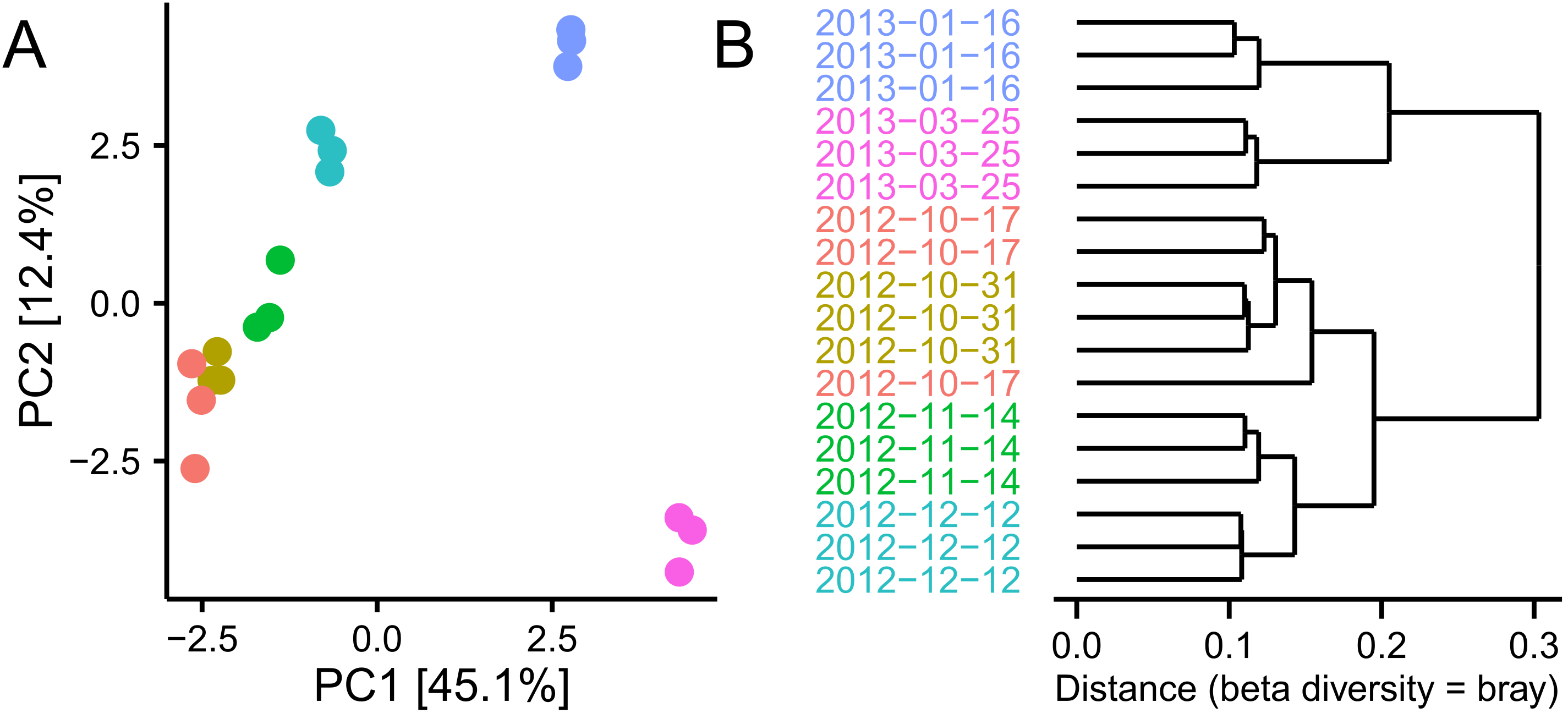

Supplement: S1 Fig — (A) PCA analysis of square root transformed OTU abundances. (B) Hierarchical clustering using Bray-Curtis dissimilarity. The variation within the biological replicates is small enough to distinguish between samples taken only weeks apart (padonis = 0.006, n = 9). (TIFF) [file pone.0132783.s001.tiff]

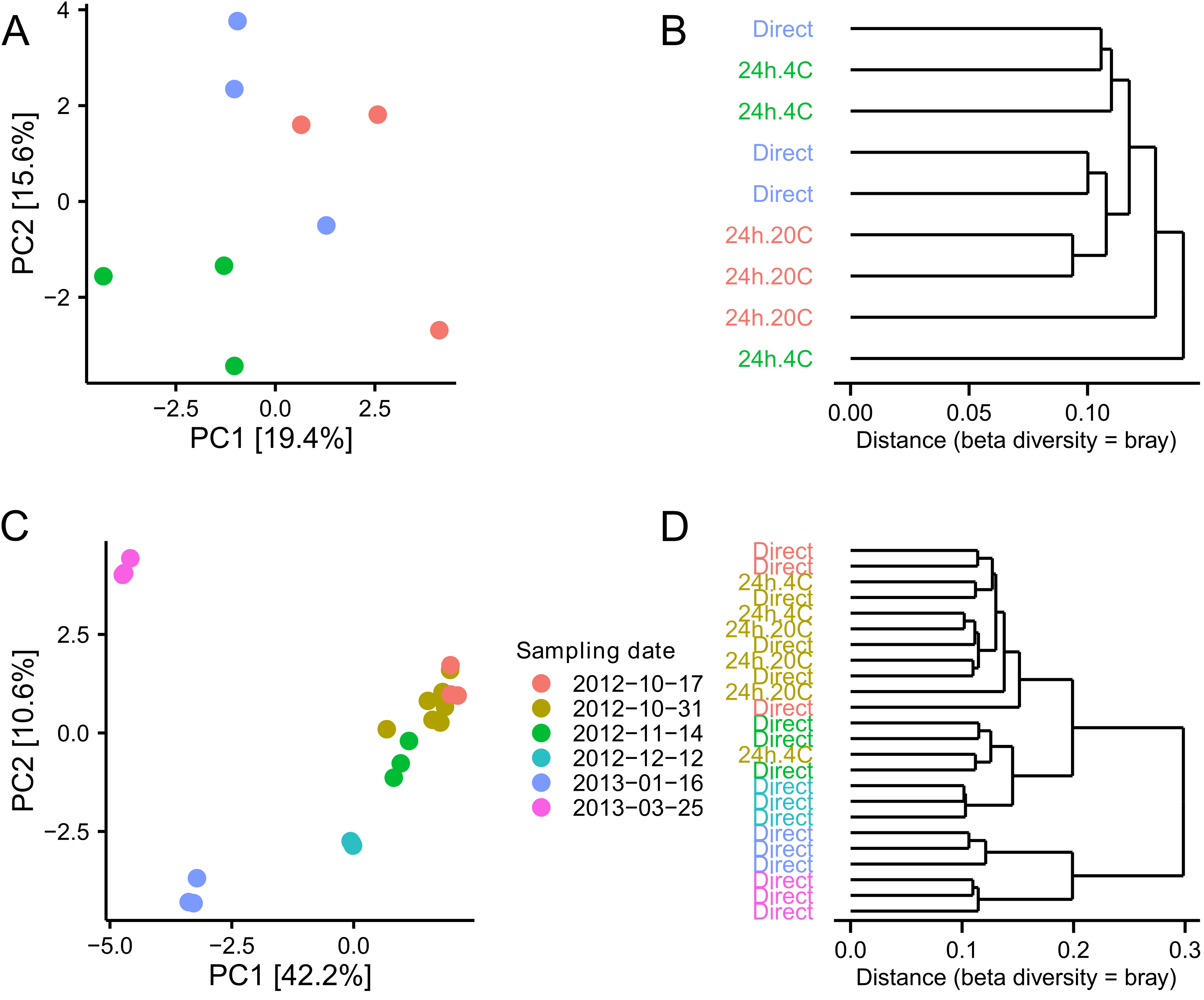

Supplement: S2 Fig — (A) PCA analysis of square root transformed OTU abundances. (B) Hierarchical clustering using Bray-Curtis dissimilarity. (C and D) The effect of storage seen in the context of the time series samples. While there was a significant effect of the short-term storage methods (padonis = 0.01, n = 9) on the overall community composition, it was small compared to the variation between samples months apart, but could potentially influence the conclusions drawn from samples within a weekly time frame. (TIFF) [file pone.0132783.s002.tiff]

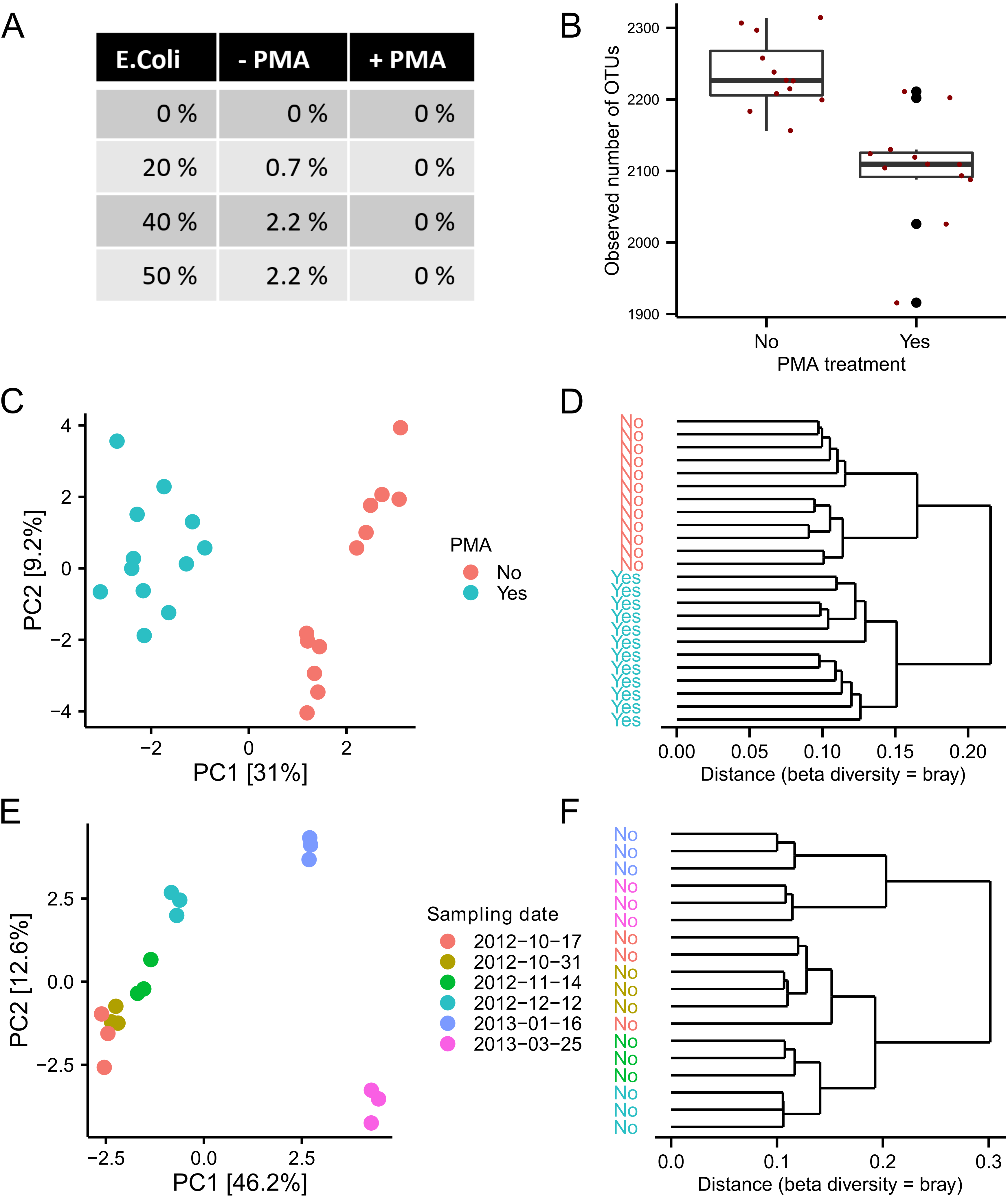

Supplement: S3 Fig — (A) PMA treatment removed all spiked-in DNA from E. coli. In the subsequent analysis, E.coli OTUs were removed and samples subsampled to the same number of sequences (17000) to facilitate comparisons using the whole dataset. (B) Impact of PMA tratment on the observed number of OTUs (pt.test = 7.2e-5, n = 24). (C) PCA analysis of square root transformed OTU abundances. (D) Hierarchical clustering using Bray-Curtis dissimilarity. The PMA treatment significantly changed the overall community profile (padonis = 0.001, n = 24). (E and F) The effect of PMA treatment seen in the context of the time series samples. (TIFF) [file pone.0132783.s003.tiff]

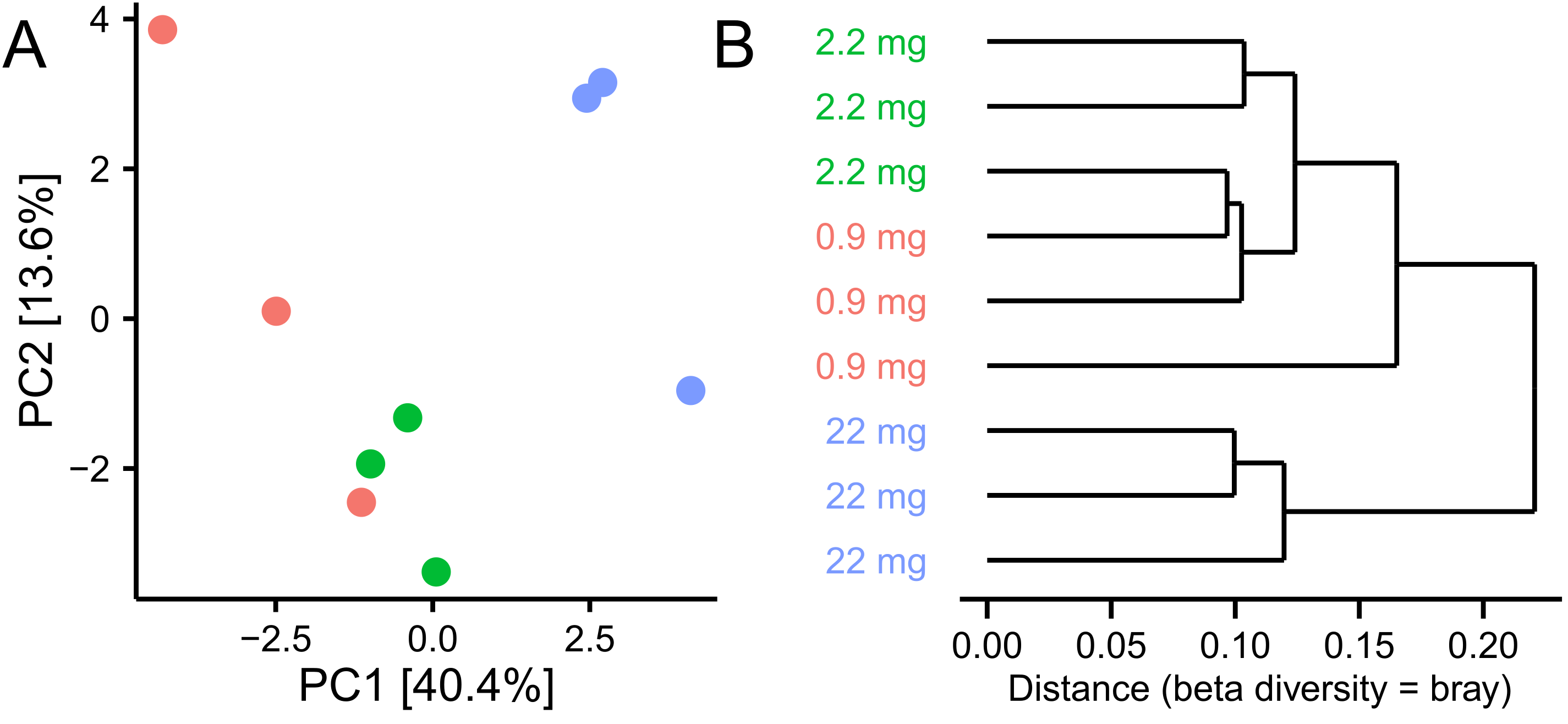

Supplement: S4 Fig — (A) PCA analysis of square root transformed OTU abundances. (B) Hierarchical clustering using Bray-Curtis dissimilarity. (TIFF) [file pone.0132783.s004.tiff]

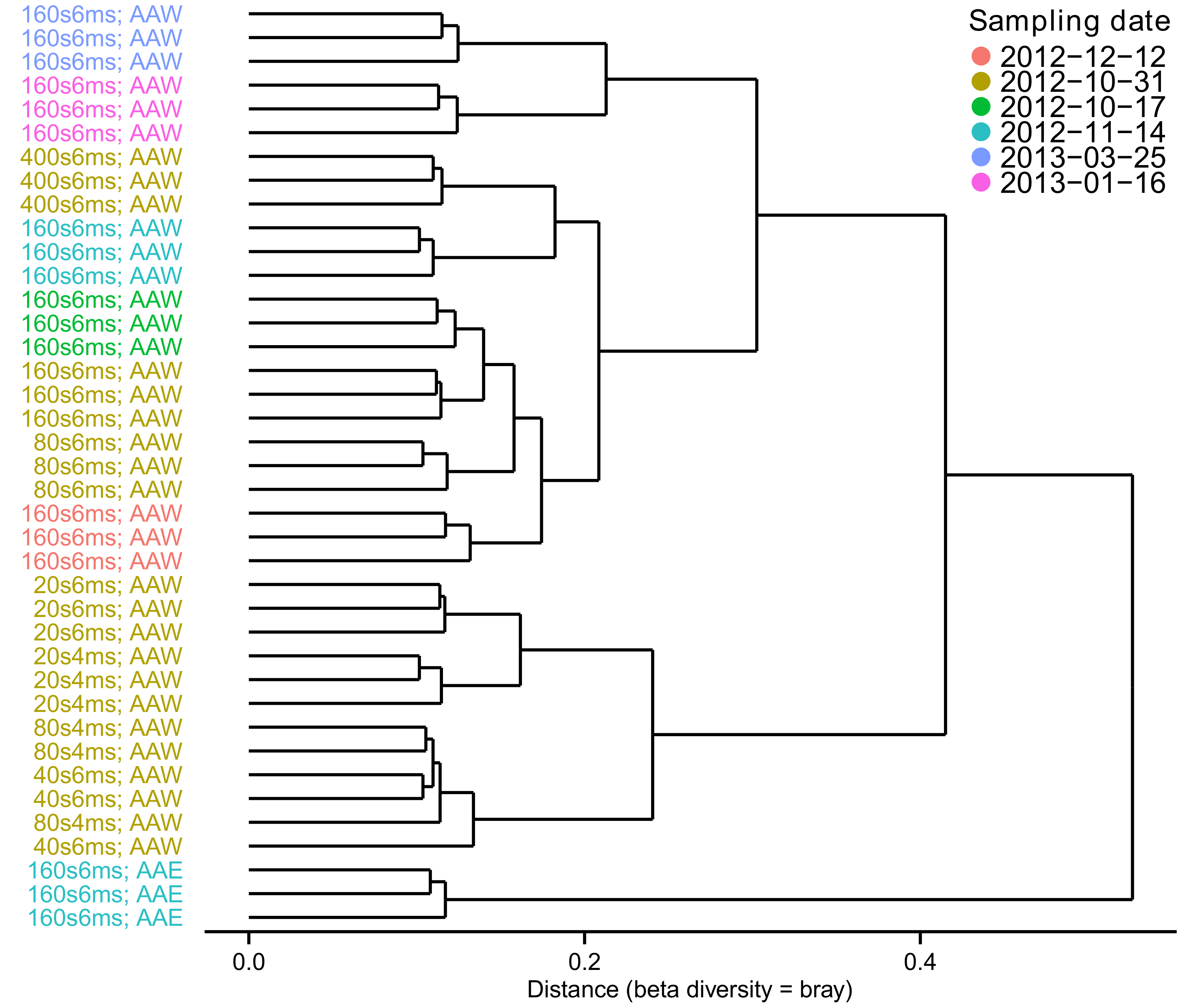

Supplement: S5 Fig — While the resolution is lost within months using different bead beating settings, the two different WWTPs can still be separated (AAW and AAE). (TIFF) [file pone.0132783.s005.tiff]

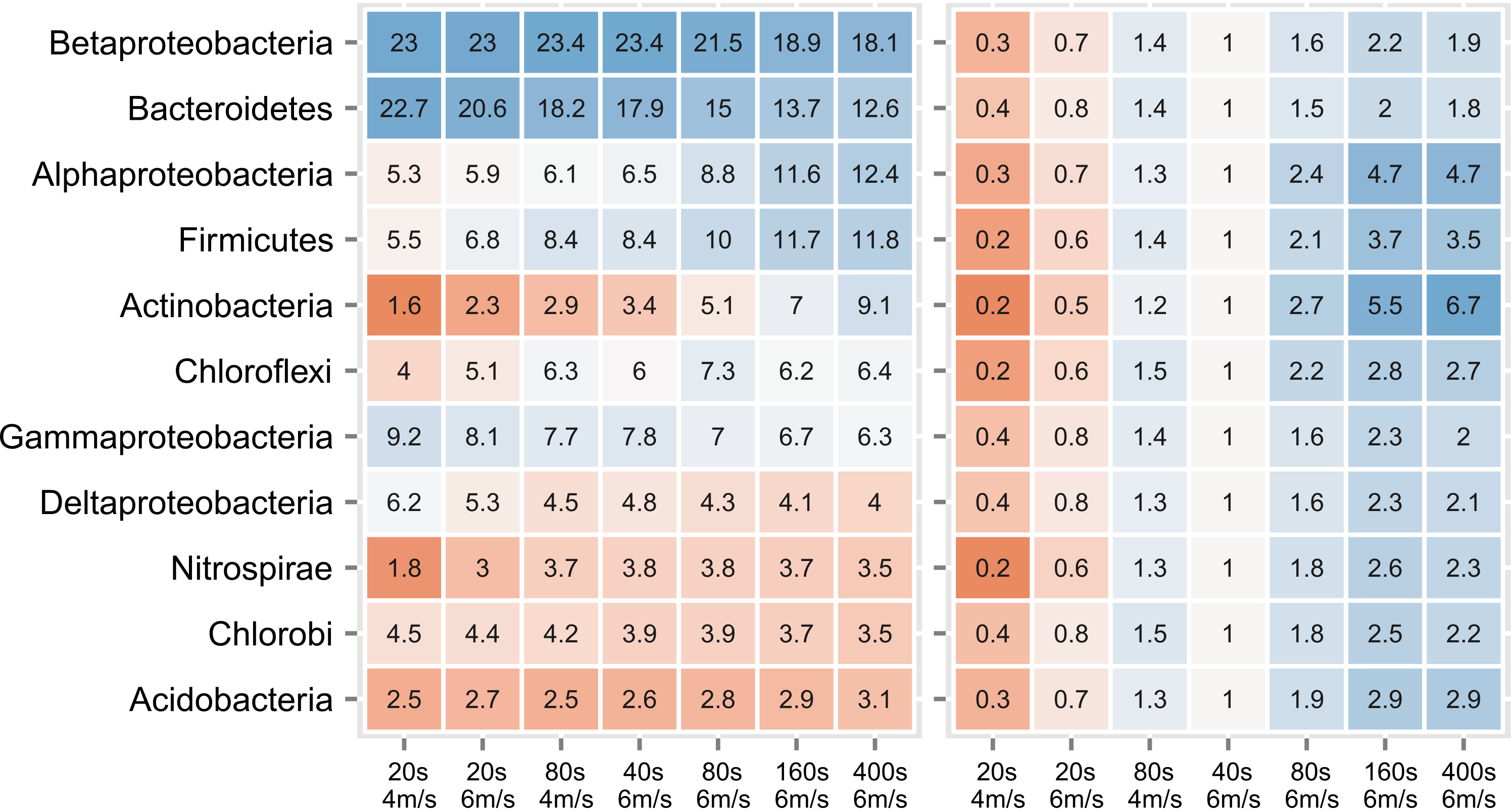

Supplement: S6 Fig — Absolute abundance was calculated by accounting for DNA yield and then normalised to the standard bead beating setting (40s at 6 m/s) to facilitate comparison between phyla (Proteobacteria are show at class level). (TIFF) [file pone.0132783.s006.tiff]

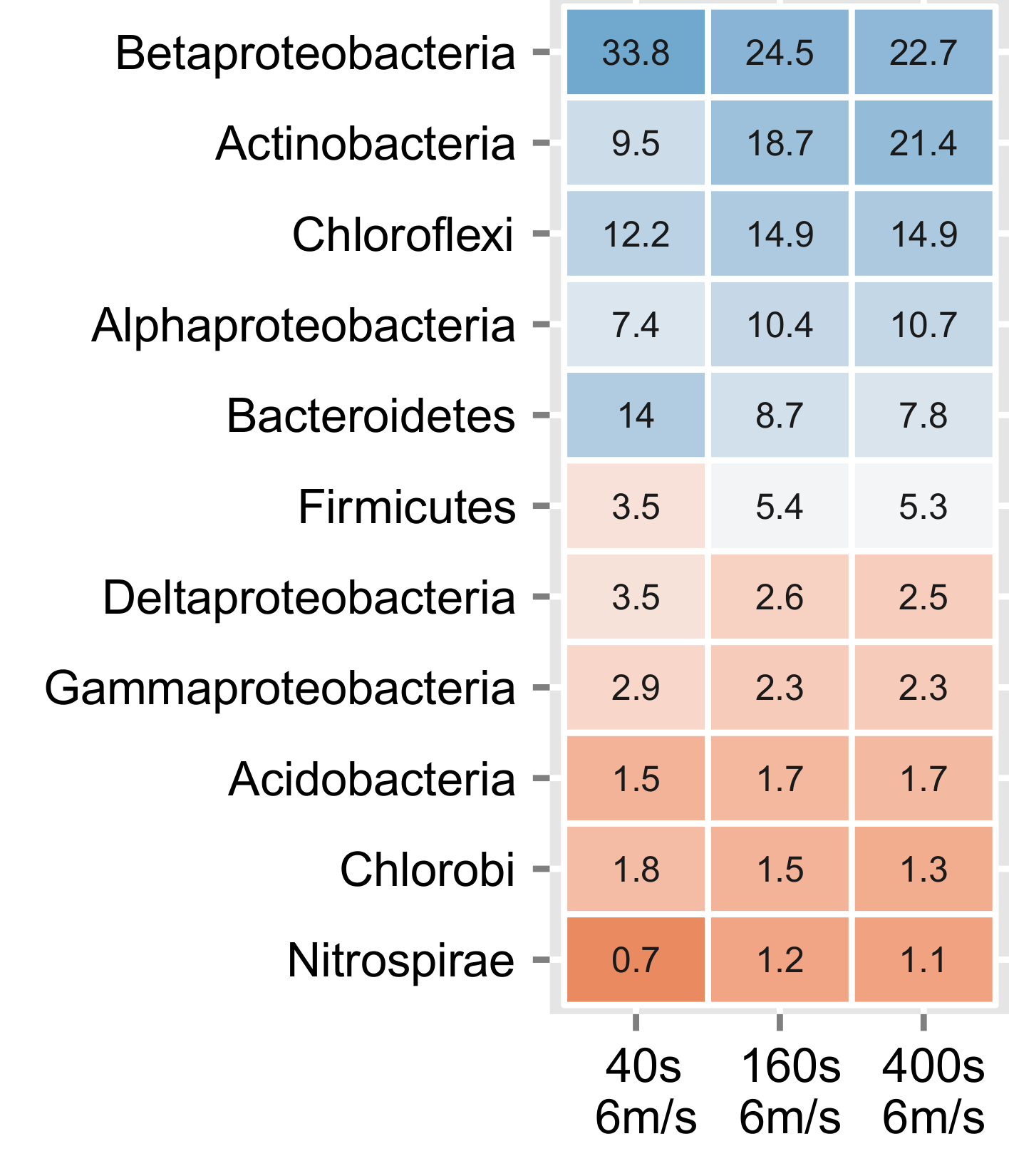

Supplement: S7 Fig — (TIFF) [file pone.0132783.s007.tiff]

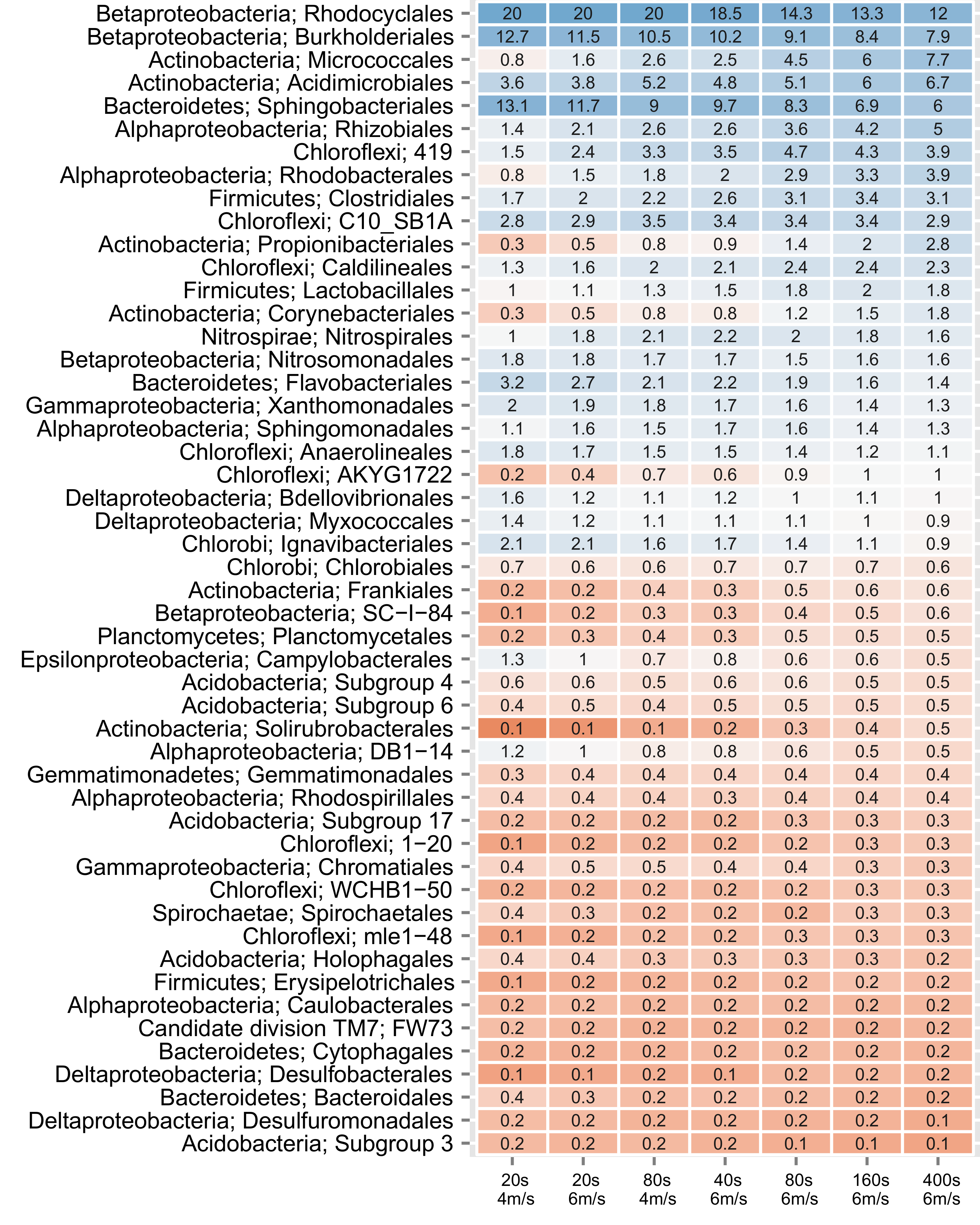

Supplement: S8 Fig — Both phylum and order level taxonomic classifications are shown (Proteobacteria are shown using classes instead). (TIFF) [file pone.0132783.s008.tiff]

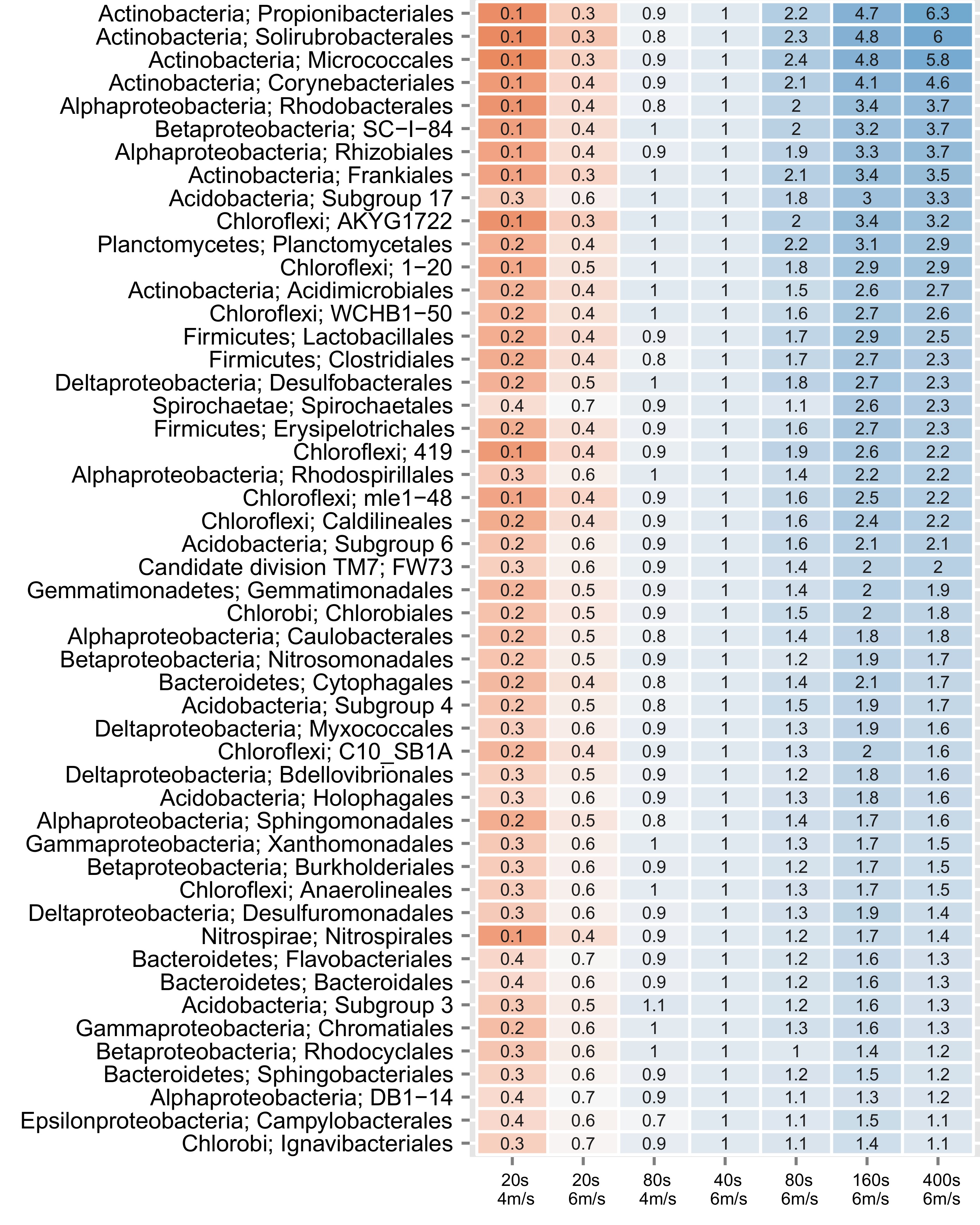

Supplement: S9 Fig — Absolute abundance was calculated by accounting for DNA yield and then normalised to the standard bead beating setting (40 s at 6 m/s) to facilitate comparison between groups. Both phylum and order level taxonomic classifications are shown (Proteobacteria are shown using classes instead). (TIFF) [file pone.0132783.s009.tiff]

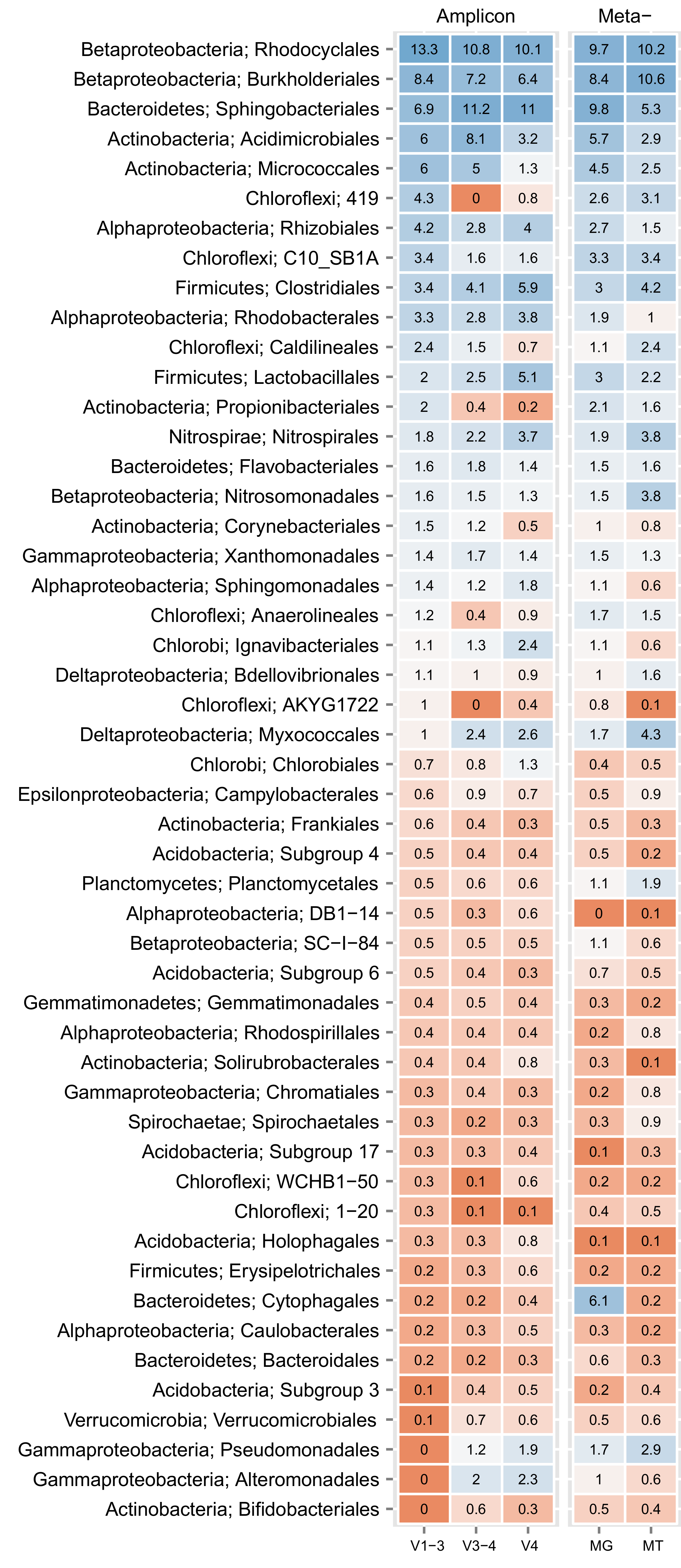

Supplement: S10 Fig — Both phylum and order level taxonomic classifications are shown (Proteobacteria are shown using classes instead). MG = Metagenome; MT = Metatranscriptome. (TIFF) [file pone.0132783.s010.tiff]
