# Supplementary material for: Back to Basics – The Influence of DNA Extraction and Primer Choice on Phylogenetic Analysis of Activated Sludge Communities
Source: PLoS One. 2015 Jul 16;10(7):e0132783. doi: 10.1371/journal.pone.0132783 (PMC4504704; doi:10.1371/journal.pone.0132783)
Supplement: S1 File — (PDF) [file pone.0132783.s011.pdf]

S1 File. Quantification limit was 0.5%. When 0.2% is indicated, it indicates that they were present but below 0.5%. 0 means none were found. Average values are from Nielsen et al., 2010. ND: Not Determined.

Functional group	Probe name	Target	Phylum	Class	Reference	Aalborg West	Average Value (%)	Range (%)
AOB	Nso190	Betaproteobacterial AOB*	Proteobacteria	Betaproteobacteria	Mobarry et al., 1996	2.5	3.8	1.2-8.2
	Nsv443	*Nitrosospira* spp.			Mobarry et al., 1996	0	1.4	0.2-3.2
	Nsm156	*Nitrosomonas* spp., *Nitrosococcus mobilis*			Mobarry et al., 1996	1.2	1.6	0.2-3.8
NOB	Ntspa662	Genus *Nitrospira**	Nitrospirae	Nitrospira	Daims et al., 2001	3.4	3.1	0.2-5.7
	Ntspa1431	Sublineage I of the genus *Nitrospira*			Maixner et al., 2006	0.3	1.6	0.2-2.5
	Ntspa1151	Sublineage II of the genus *Nitrospira*			Maixner et al., 2006	0.3	1.4	0.2-3.9
Denitrifiers	Curvi997 (Aqs997)	*Curvibacter* (former *Aquaspririllum*)*	Proteobacteria	Betaproteobacteria	Thomsen et al., 2004	5.3	7.1	1.5-14.1
	ZRA23a (ZRA, ZOGLO647)	Most members of *Zoogloea* lineage, not *Z. Resiniphila**			Rossello-Mora et al., 1995	0.5	2.1	0.2-5.2
	Azo644 (AZA645)	Most members of the *Azoarcus* cluster*			Hess et al., 1997	3.2	3.1	0.8-9.3
	Thau646	*Thauera**			Lanjoie et al., 2000	3.8	3.1	0.2-6.2
	G Rb	*Rhodobacter, Roseobacter**		Alphaproteobacteria	Giuliano et al., 1999	2.2	1.2	0.2-3.4
PAO/Denitrifiers	PAOmix (PAO462, PAO651, PAO846)	Most Accumulibacter*		Betaproteobacteria	Crocetti et al., 2000	1.6	3.6	1.0-10.8
	Acc-I-444	Accumulibacter – clade IA and others			Flowers et al., 2008	ND	1.5	0.2-4.6
PAO	Acc-II-444	Accumulibacter – clade IIA, IIC, IID		Betaproteobacteria	Flowers et al., 2008	ND	1.1	0.2-3.2
	Actino-221	Some *Tetrasphaera*	Actinobacteria	Actinobacteria	Kong et al., 2005	ND	4.3	0.8-9.9
	Actino-658	Some *Tetrasphaera*			Kong et al., 2005	ND	4.1	1.0-10.8
	Tet1-266	*Tetrasphaera* clade I*			Nguyen et al., 2011	8.5	ND	ND
	Tet2-892, Tet2-174	*Tetrasphaera* clade 2*			Nguyen et al., 2011	5.8	ND	ND
	Tet3-654	*Tetrasphaera* clade 3*			Nguyen et al., 2011	3.9	ND	ND
GAO	GBmix	Most *Competibacter**	Proteobacteria	Gammaproteobacteria	Kong et al., 2002	0.3	1	0-8.0
Protein hydrolysers	SAP-309	Saprospiraceae*	Bacteroidetes	Sphingobacteria	Schauer and Hahn 2005	3.5	6.1	1.2-12.2
Fermenters	Str	*Streptococcus* spp.*	Firmicutes	Bacilli	Trebesius et al., 2000	1.3	2.3	0.2-6.7
Filamentous bacteria	G123 T	*Thiothrix eikelboomii, T. nivea, T. unzii, T. fructosivorans, T. defluvii, T. Eikelbloom* Type 021N group I, II, III*	Proteobacteria	Gammaproteobacteria	Kanagawa et al., 2000	0.3	0.9	0.2-4.5
	MPAmix	*Candidatus* Microthrix parvicella, *Candidatus* Microthrix calida*	Actinobacteria	Actinobacteria	Erhart et al., 1997; Levantesi et al., 2006	4.2	5.6	0.2-15.4
	CFXmix (GNSB941, CFX1223)	Phylum Chloroflexi*	Chloroflexi		Gieseke et al., 2001	10.0	10.2	2.4-22.3
	TM7905	Candidate division TM7*	Candidate division TM7		Hugenholtz et al., 2001	3.6	4.9	0.9-12.3
	HHY-654	Several *Haliscomenobacter* species among others the type strain	Bacteroidetes	Sphingobacteria	Kragelund et al., 2008	2.2	1.9	0.2-6.3

* Data used for calculating summed abundance. Data without an asterisk is covered by a broader probe.

References

Crocetti GR., Hugenholtz P, Bond PL, Schuler A, Keller J, Jenkins D, Blackall LL. Identification of polyphosphate-accumulating organisms and design of 16S rRNA-directed probes for their detection and quantitation. Appl Environ Microbiol. 2000; 66: 1175-1182.

Daims H, Nielsen JL, Nielsen PH, Schleifer KH, Wagner M. In situ characterization of Nitrospira-like nitrite- oxidizing bacteria active in wastewater treatment plants. Appl Environ Microbiol. 2001; 67 :5273-5284.

Erhart R, Bradford D, Seviour RJ, Amann R, Blackall LL. Development and use of fluorescent in situ hybridization probes for the detection and identification of 'Microthrix parvicella' in activated sludge. Systematic and Applied Microbiol. 1997; 20: 310-318.

Flowers J, He S, Carvalho G, Peterson SB, Lopez C, Yilmaz S, et al. Ecological differentiation of Accumulibacter in EBPR reactors. In: Proceedings of the Water Environment Federation, WEFTEC. 2008; 12: 31-42.

Gieseke A, Purkhold U, Wagner M, Amann R, Schramm A. Community structure and activity dynamics of nitrifying bacteria in a phosphate-removing biofilm. Applied and Environmental Microbiololy. 2001; 67: 1351-1362.

Giuliano L, De Domenico M, De Domenico E, Hofle MG, Yakimov MM. Identification of culturable oligotrophic bacteria within naturally occurring bacterioplankton communities of the Ligurian sea by 16S rRNA sequencing and probing. Microbial Ecology. 1999; 37: 77-85.

Hess A, Zarda B, Hahn D, Haner A, Stax D, Hohener P, Zeyer J. In situ analysis of denitrifying toluene- and m-xylene-degrading bacteria in a diesel fuel-contaminated laboratory aquifer column. Appl Environ Microbiol. 1997; 63: 2136-2141.

Hugenholtz P, Tyson GW, Webb RI, Wagner AM, Blackall LL. Investigation of Candidate division TM7, a recently recognizedmajor lineage of the domain bacteriawith no known pure-culture representatives. Appl Environ Microbiol. 2001; 67: 411-419.

Kanagawa T, Kamagata Y, Aruga S, Kohno T, Horn M, Wagner M. Phylogenetic analysis of and oligonucleotide probe development for Eikelboom type 021N filamentous bacteria isolated from bulking activated sludge. Appl Environ Microbiol. 2000; 66: 5043-5052.

Kong YH, Beer M, Rees GN, Seviour RJ. Functional analysis of microbial communities in aerobiceanaerobic sequencing batch reactors fed with different phosphorus/ carbon (P/C) ratios. Microbiology-Sgm 2002; 148: 2299-2307.

Kong Y, Nielsen JL, Nielsen PH. Identity and ecophysiology of uncultured actinobacterial polyphosphate- accumulating organisms in full-scale enhanced biological phosphorus removal plants. Appl Environ Microbiol. 2005; 71: 4076-4085.

Kragelund C, Levantesi C, Borger A, Thelen K, Eikelboom D, Tandoi V, et al. Identity, abundance and ecophysiology of filamentous bacteria belonging to the Bacteroidetes present in activated sludge plants. Microbiology. 2008; 154: 886-894.

Lajoie CA, Layton AC, Gregory IR, Sayler GS, Taylor DE, Meyers AJ. Zoogloeal clusters and sludge dewatering potential in an industrial activated-sludge wastewater treatment plant. Water Environment Research. 2000; 72: 56-64.

Levantesi C, Rossetti S, Thelen K, Kragelund C, Krooneman J, Eikelboom D, et al. Phylogeny, physiology and distribution of 'Candidatus Microthrix calida', anew Microthrix species isolated from industrial activated sludge wastewater treatment plants. Environ Microbiol. 2006; 8: 1552-1563.

Maixner F, Noguera DR, Anneser B, Stoecker K, Wegl G, Wagner M, Daims H. Nitrite concentration influences the population structure of Nitrospira-like bacteria. Environ Microbiol. 2006; 8: 1487-1495.

Mobarry BK, Wagner M, Urbain V, Rittmann BE, Stahl DA. Phylogenetic probes for analyzing abundance and spatial organization of nitrifying bacteria. Appl Environ Microbiol. 1996; 62: 2156-2162.

Nguyen HTT, Le VQ, Hansen AA, Nielsen JL, Nielsen PH. High diversity and abundance of putative polyphosphate-accumulating *Tetrasphaera*-related bacteria in activated sludge systems. FEMS Microbiol Ecol. 2011; 75: 256-67.

Rossello-Mora RA, Wagner M, Amann R, Schleifer KH. The abundance of Zoogloea ramigera in sewage treatment plants. Applied and Environ Microbiol. 1995; 61: 702-707.

Schauer M, Hahn MW. Diversity and phylogenetic affiliations of morphologically conspicuous large filamentous bacteria occurring in the pelagic zones of a broad spectrum of freshwater habitats. Appl Environ Microbiol 2005; 71: 1931-1940.

Thomsen TR, Nielsen JL, Ramsing NB, Nielsen PH (2004). Micromanipulation and further identification of FISH-labelled microcolonies of a dominant denitrifying bacterium in activated sludge. Environ Microbiol. 2004; 6: 470-479.

Trebesius K, Leitritz L, Adler K, Schubert S, Autenrieth IB, Heesemann J. Culture independent and rapid identification of bacterial pathogens in necrotising fasciitis and streptococcal toxic shock syndrome by fluorescence in situ hybridisation. Medical Microbiology and Immunology. 2000; 188: 169-175.
